# Cardiovascular effects of fingolimod: A review article

**Published:** 2014-07-04

**Authors:** Mohaddeseh Behjati, Masoud Etemadifar, Morteza Abdar Esfahani

**Affiliations:** 1 Department of Cardiology, School of Medicine, Cardiovascular Research Center AND Heart Failure Research Center, Isfahan Cardiovascular Research Institute, Isfahan University of Medical Sciences, Isfahan, Iran; 2 Department of Neurology, School of Medicine, Isfahan University of Medical Sciences, Isfahan, Iran; 3 Department of Cardiology, School of Medicine, Isfahan University of Medical Sciences, Isfahan, Iran

**Keywords:** Cardiovascular side-effects, Fingolimod, Multiple Sclerosis

## Abstract

Multiple sclerosis (MS) is a chronic auto-immune disease. Most therapeutic strategies for treatment of this disease direct immune modulation and control of inflammatory processes. First-line therapeutic agents showed moderate efficacy and frequent side-effects with moderate efficacy in trials. Their parental administration and limited long-term adherence restrict their efficacy compared with second-line therapies. Fingolimod as a second-line therapeutic agent has been shown to reduce annualized relapse rate, risk of disability progression and inflammatory activity of relapsing MS. Safety and efficacy FTY720: Safety and efficacy issues are the main metrics for judgment of drug efficacy. In this article, we focus on cardiovascular effects of FTY720 treatment. Effect of FTY720 on rate and rhythm, impact of FTY720 on endothelial cells, its atheroprotective effects, its effects on cardiac transplantation outcomes, vascular complications of FTY720, effects of FTY720 on endocrine functions and interaction of FTY720 with cardioactive agents are explained in this review article.

## Introduction

Multiple sclerosis (MS) is considered as a chronic auto-immune disease.^1^^,^^2^ Therapeutic strategies direct immune modulation and control of inflammatory processes. Until now, five first-line and two second-line therapeutic agents are available.^3^ First-line therapies as interferon beta-1 and glatiramer acetate showed moderate efficacy and frequent side-effects with moderate efficacy in trials and due to their parental administration, limited long-term adherence consequently restrict their efficacy compared with second-line therapies as fingolimod and natalizumab.^3^^,^^4^ Thus, emergence of novel drugs is seriously needed.

Drug development for MS is a challenging field of science. Fingolimod 2-amino-2-[2-(4-octylphenyl) ethyl]propane diolhydrochloride) also known as FTY720 or Gilenya, is a Food & Drug Administration-approved agent for treatment of relapsing remitting MS (RRMS).^4^^-^^6^ This compound derived from myriocin, a component isolated from the culture filtrate of natural product ascomycete *Isaria sinclairii*.^7^ This licensed drug offers a far more convenient route of administration. This sphingosine-like synthetic analog sequesters auto-reactive thymocytes and lymphocytes from lymph nodes through its similarity to gatekeeper sphingosine-1-phosphate (S1P). This fungal metabolite is oral super-agonist of pleiotropic S1P receptor (S1PR), which blocks various signaling pathways mediated by interaction of this endogenous lysophospholipid and its receptor.^8^ S1P has five major subtypes (S1P1-5), in which each of them has a special pattern of expression.^9^ FTY720 is a non-selective agonist of S1PR.^10^ Marked S1P1 receptor internalization occurs upon treatment by FTY720. S1P is crucially involved in vascular barrier function, thus regulate inflammation, coagulation, vascular homeostasis, angiogenesis, tumor metastasis, and atherosclerosis.^11^ By blocking S1PR on the surface of lymphocytes and cells in central nervous system, including glial cells and neurons; it reduces annualized relapse rate (ARR), risk of disability progression, tissue damage, and brain atrophy and inflammatory activity of relapsing MS.^12^

***Pharmacokinetics and Pharmacodynamic Pathway of FTY720***

FTY720 is a pro-drug, which is reversibly phosphorylated to its biologically active moiety phospho-FTY720 (FTY720-P) by sphingosine kinase (SphK2) within minutes to construct a nonselective S1PR agonist.^13^^,^^14^ By CYP4F isoenzyme, FTY720 is irreversibly metabolized to its metabolites.^15^ Fingolimod in its phosphorylated form binds to four of the five S1PRs (except S1PR2).^16^ It has a high blood bioavailability by its oral administration and has a low inter-individual coefficient of variation.^17^ It should be used with caution when combined with class Ia and III anti-arrhythmic agents, beta-blockers, and ketoconazole.^19^

***Interaction of FTY720 with Cardioactive Agents***

Interaction between FTY720, atenolol and diltiazem is unlikely. A similar acute negative chronotropic effect is elicited using 5-mg single dose of FTY720 and atenolol alone. Addition of FTY720 to atenolol leads to moderate further reduction of heart rate (15% lower). Addition of a calcium channel blocker to FTY720 was not associated with further lowering of heart rate compared with alone. FTY720 did not alter antihypertensive effects of atenolol. Stronger negative chronotropic response to FTY720 alone (<50 bpm) was parallel with no or further decrease heat rate when combined with atenolol. The negative chronotropic effects of FTY720, was stronger than diltiazem alone.^15^ P-glycoprotein activity is reduced by FTY720/FTY720P, which leads to the increase in verapamil and loperamide uptake.^16^

***Safety and Efficacy FTY720***

Safety and efficacy issues are the main metrics for judgment of drug efficacy. Thus, the aim of this review article is to prepare information about the advantages and disadvantages of FTY720 regarding cardiovascular system. In this narrative review article, we have searched articles published on the impacts of FTY720 on cardiovascular system using search keywords of MS, cardiovascular system, FTY720, side-effects, and complications.

FTY720 has been shown to be superior to placebo and INF-1B in phase II trials.^19^ In a trial, assessing effects of daily oral therapy in MS (FREEDOMS) and comparing FTY with intramuscular interferon beta-1a administered once weekly [Trial Assessing injectable interferon vs. FTY720 Oral in Rapidly progressing MS (TRANSFORMS) are two phase III studies on FTY720.^20^ Among side-effects of fingolimod, fatigue, nasopharyngitis, and influenza have been reported more frequent than others.^21^ Other reported side-effects are as headache, fever, chills, muscle pain, swelling in hands/feet, loss of appetite, susceptibility to infection, higher risk of herpetic infections, itching, mild hair loss, eczema, itchy rash, diarrhea, back pain, cough, dizziness, nausea, vomiting sensitivity to light, numbness, tingling, weight loss, weakness, respiratory disturbances as wheeze and dyspnea, liver enzyme elevation, jaundice clay-colored stool, sores in the throat and mouth, sinusitis, bronchitis, depression, vision problems as blurred vision, and macular edema.^22^^-^^25^ It passes from breast milk with unknown effects on infants of breastfeeding women. Indeed, FTY720 induce fetal harm as teratogenicity and embryopathy in animal pregnancy (category C).^26^ The effects of FTY720 on human pregnancy are not yet known. Thus, women at childbearing age under treatment with fingolimod should apply tight birth control strategies. The safety of FTY720 for pediatric age is not well-demonstrated. In addition to its effects on lymphocyte migration, there is increasing evidence for cardiovascular side-effects of fingolimod. Since the use of FTY720 in clinical practice became limited partly due to concerns for cardiac effects, hereby, we focus on cardiovascular effects of FTY720 treatment.

***Effects of FTY720 on Rate and Rhythm***

S1PR agonists regulate cardiovascular functions; therefore, FTY720 targets cardiovascular system. Indeed, S1P is involved in embryonic development of cardiovascular system.^27^ In phase I and II clinical trials, fingolimod was known to be a safe agent from cardiovascular aspects, except for acute modest, but significant transient and dose-dependent bradycardia.^28^ Symptomatic bradycardia, which occurs in about 0.5% of cases is most often self-limiting.^29^ Rarely, occurrence of fatal bradyarrythmia using FTY720 has also been reported.^30^ These effects have also been observed in healthy volunteers.^31^ The decrease in mean nadir heart rate is up to 10 bpm after first does without incremental decrease in heart rate after day two of drug.^4^ Most often, heart rate and conduction normalized in 24 h after first Reduction of heart rate occurs maximally within the first 4–6 h after the first does with consequent attenuation over time by continues consumption. 

The magnitude of bradycardia did not increase with repeated dosing despite of increase in its blood concentration^3^ and with continued treatment, heart rate returns toward baseline values.^11^^,^^15^ Following treatment initiation, an approximate 10 bmp decrease in heart rates. In the first 12 h post-dose, a dose-dependent decrease in heart rate was evident, in which the adjusted mean heart rate was as follow: 73.6 bpm for Hp4: 0.5 mg (adjusted mean treatment difference: 7.9 bpm) and 69.6 bpm for 1.25 mg (adjusted mean treatment difference: 11.9 bpm). Decrease in heart rate was more evident in 5 h and 3 h post-dose, which persisted for 8 h and 24 h dose time interval, heart rate became similar to placebo group. Mean heart rates on the day 7, was lower for both 0.5 and 1.25 mg vs. placebo group throughout the first 24 h dose interval. The heart rates over 12 h post-dose were still approximately 10 bpm group by day 14. This side-effect has been reported to be most often asymptomatic. The mean heart rate is lower after once daily oral FTY720 compared with intravenous administration (53 bpm) due to the higher blood level of active metabolites of fingolimod by former.^15^ Fingolimod-induced bradycardia is totally reversible using atropine. Interestingly, an intravenous titration of atropine during fingolimod administration could prevent typical nadir heart rate which occurs around 4 h post-dose of fingolimod. 

The mean total dose of 1.4 mg atropine was able to reverse the bradycardia attributed to fingolimod. Continued atropine titration to the mean total of 1.9 mg, was associated with raised and maintained heart rate at lower normal limit lasting for a median 3.5 h. The therapeutic doses of atropine for reversal of FTY720-related bradycardia are within the normal therapeutic range of atropine for the treatment of acute asymptomatic and sinus bradycardia. Acute effects of atropine would occur at the first 24 h after FTY720 administration.^32^ FTY720 has been shown to have non-significant effects on circadian rhythm, oxygen exchange, airflow and hemodynamic variables as cardiac output and systemic vascular resistance (SVR) during 14 days treatments in healthy volunteers.^15^^,^^34^ Benign atrioventricular block (type I or Wenckebach) has reported using FTY720.^32^ An approximate, 8–10 ms increase in PR-interval has been reported using FTY720, without any change on QRS or QT intervals Despite of slowing atrioventricular conduction, the incidence of Mobitz type II atrioventricular blocks and 2:1 atrioventricular block is respectively. Conduction abnormalities showed to regress during the time and in therapeutic doses, higher degrees of the block were not seen.^29^

S1PRs are not only expressed on lymphocytes, but also expressed on the surface of atrial myocardial cells. All S1PRs (S1P1-3) in heart are stimulated using S1P which leads to activation of (Gi, Gq and G12/13) but only S1P1 and S1P3 receptors are activated using FTY720, which leads to activation of Gi.^30^^,^^31^ Thus, the underlying mechanism of bradycardia is due to the activation of inwardly rectifying Gà1-protein-regualted potassium channel (GIRK/IKACh) channels in atrial myocytes and endothelial cells). The function of acetylcholine-regulated potassium channel (KACh) is stimulated by S1P. S1PR regulates heart rate through binding to its receptors on the surface of atrial myocytes.^33^ This inhibited cardiac pacemaker activity is similar to the vagally-mediated cardiac effects through the same G protein-gated potassium channel with different pathway FTY720 induces dephosphorylation of cTnI in ventricular myocytes. Repeated dosing of fingolimod leads to S1RPs internalization and cessation of signaling.^34^

***Effects of FTY720 on Endothelial Cells***

Due to the tissue-specific arrangement of S1PR subtypes, diverse differential cardiovascular effects are not unexpected. S1P1/3 are the major mediators of S1P-related actions on cardiac microvascular endothelial cells.^30^ Specifically, subtypes of S1P and S1P3 are both expressed on the surface of endothelial cells and are involved in vascular stabilization.^34^^,^^35^ Identification of S1PR involvement in endothelial cell differentiation for the first time, hints to the occurrence of cardiovascular side effects by fingolimod.^36^^,^^37^ S1P is participated in endothelial cell proliferation, differentiation, migration, and survival.^30^ S1P stimulates functional capacity of endothelial progenitor cells.^38^^,^^39^ It also affects smooth muscle or bone marrow cells through activation of G protein coupled S1PRs. S1P is involved in maintenance of vascular endothelial barrier integrity and angiogenic homeostasis.^30^ Activation of S1P1 promotes angiogenesis, whereas stimulation of S1P3 impairs barrier function.^39^ FTY720 acts on endothelial cells and is involved in the preservation of vascular integrity through enhancement of adhesion junctional assembly and endothelial barrier functions.^40^

FTY720 might act as an agonist for S1P1 and a functional antagonist for S1P3, in order to keep the balance between angiogenesis and microvascular barrier function.^30^ The agonist and antagonist effects of FTY720 are exerted through up-regulation of S1P1 and translocation of S1P3.^32^ Down-regulated S P1 and translocation of S1P3 from nuclear to the membrane is in parallel with increased cardiac microvascular permeability and its consequents as pathologic angiogenesis, inflammation, and pathologic conditions as sepsis, tumor growth, acute lung injury, hypoxia and microvascular complications seen in diabetes.^33^^,^^41^^,^^42^ It has been suggested that fingolimod assist in the preservation of endothelial integrity of vasculature.^32^

Fingolimod-induced endothelium dependent vasodilatation in the mouse aorta has been attributed to the potent activation of Akt/eNOS/NO pathway.^37^ Fingolimod significantly impair flow-mediated dilation. Arterial vasodilatory function improves by discontinuation of FTY720.^33^ Through beneficial interaction with PDGF and VEGF, FTY720 exhibited good anti-tumor and anti-angiogenesis properties.^39^ By barrier stabilization, diminishing invasion, migration and capillary tube formation in human umbilical vein endothelial cells at very low doses of FTY720, tumor metastasis has suppressed in vitro.^43^ Since, S1RPs are expressed on the surface of the alveolar epithelium and lung capillary endothelium, which are involved in regulation of alveolar-capillary barriers and smooth-muscle cell tone and hypertrophy, fingolimod seems to reduced pulmonary capacity.^14^ In FREEDOMS and TRANSFORMS trials administration of 0.5–1.25 mg of FTY720 has been associated with minor changes on forced expiratory volume in 1 s (FEV1) after 1 month therapy, which remained stable thereafter. Other parameters of pulmonary function test were unchanged.^31^ A dose-dependent decrease in FEV1 was seen on day one with methacholine challenge test (methacholine dose of 0.25–25 mg). No bronchodilatory response to inhaled albuterol was also seen. These data suggest that fingolimod dose is ineffective on increased bronchial hyperreactivity and no paradoxical increase in airway resistance to albuterol challenge is elicited by fingolimod treatment. Dyspnea and asthma attacks were seen more frequently by 5.0 rather than 1.25 mg fingolimod. Fingolimod, larger reductions in FEV1 from baseline were seen compared with placebo (8.8% vs. 1.9%).^44^ It should be noted that and chemically modified from a Chinese herb traditionally used for the treatment of asthma.^45^ However, this was transient and limited to the first few weeks of administration. Overall, pulmonary effects of fingolimod are mild and with minimal clinical impact at the dose applied for treatment of MS.^45^

***Atheroprotective Effects of FTY720***

Fingolimod has been shown to decrease atherosclerotic wall changes in apolipoprotein E-deficient mice.^32^ FTY diminished atherosclerosis plaque volume and its macrophage and collagen content in mice with hypercholesterolemic diet.^46^ Short-term low-dose oral FTY720 significantly reduced early development of atherosclerosis in mice.^47^ In these mice, blood concentration of anti-inflammatory cytokines was increased. FTY720 inhibits sphingosine kinase independent from S1PRs, which leads to the cell apoptosis via modulation of ceramide sphingosine-S1P rheostat.^48^^,^^49^ Indeed, some anti-angiogenic effects of FTY720 have been reported thorough blocking S1PRs involved in recruitment of mural cells during angiogenesis. Since S1PR3 is predominantly expressed on the surface of adult rat VSMCs, FTY720 can inhibit migration of VSMCs. S1P-blokage using FTY720 weakly affects VSMCs spatial organization. Combined inhibition of PDGFR and S1PR1/3 completely abolish network forming capabilities of VSMCs, which make them good candidates for anti-angiogenic and anti-atherosclerotic treatments due to the fundamental roles of VSMC migration in these processes.^49^ Indeed, FTY720 has been reported as a cardio-protective agent through reliving either tachyarrhythmia or bradyarrhythmia induced by cardiac ischemia/reperfusion injuries.^50^ It improves recovery of cardiac function after myocardial ischemia-reperfusion Applied FTY720 during reperfusion decreased left ventricular end diastolic pressure and vice versa increased mortality due to induction of fatal arrhythmia without reduction of infarct size. Pretreatment with FTY720 before ischemia diminished pro-arrhythmia without any effect on infarct size. Thus, FTY720 has a potential role in preconditioning and post-conditioning.^51^

The underlying mechanisms of this cardioprotection are unknown but it has been suggested that FTY720 cardioprotection pass through S1P cascade to p-21 activated kinase (Pak1), Akt cascade and inhibitory G protein Gi).^52^^,^^53^ Pak1 a Ser/The kinase downstream of small G proteins is activated in a time- and dose-dependent manner through sphingosine. Pak1 activity regulates cardiac channel activity and contractility, cytoskeletal dynamics, cell motility, growth and proliferation. FTY720 directly activate Pak in cardiac cells without primary conversion to FTY720 phosphate.^52^ Indeed, pharmacologic dose of FTY720 (10 mg/kg/day) resists load stress-induced murine hypertrophic remodeling without deterioration of the cardiac function through Pak1 activation. Significant decrease in HW/TL ratio and in mean cross sectional area was seen by FTY720 administration. 

Attenuating cardiac hypertrophy and halting transition to heart failure with preservation of cardiac function is very beneficial for prevention and treatment of cardiac hypertrophy, since according to Laplace's law limited cardiac hypertrophy might be parallel with chamber dilation and cardiac deterioration. Pak1 is a critical signaling hub in cardioprotection, which is mutually involved in limitation of excessive hypertrophic remodeling. Thus, both anti-hypertrophic and survival signals are conveyed from small GTPases to JNK pathway in cardiomyocytes by activated. The possible downstream molecule for Pak1 in this signaling path seems to be Cdc42.^54^ These data hints to the application of FTY720 as a non-toxic compound with oral bioavailability in the prevention and/or treatment of cardiac disorders in high-risk patients. In addition to beneficial effects of FTY72 in cardiac disorders, FTY720 has been shown to be promising for the treatment of ischemic stroke.^55^ In this case, protection of neurovascular unit in stroke, reduction of infarct lesion size and improvement of neurological function has been seen using FTY720. Diminished infiltration of immune cells and reduced apoptotic cell death in ischemic stroke lesions is in parallel with improved neurologic functions using FTY720 in ischemic strokes.^56^

***Effects of FTY720 on Cardiac Transplantation Outcomes***

FTY720 up-regulates different intracellular protective molecules as heat shock proteins which lead to stabilization of endothelial layer and decreased sensitivity of endothelial cells to inflammatory cytokines. This prevents organs from transmigration of activated inflammatory cells across endothelial layer and paranchymal infiltration, which consequently results in organ salvage.^59^ Thus, FTY720 has been proposed as an immunosuppressant agent for organ transplantation purposes. Rejection, infection and drug toxicity are the leading cause of morbidity and mortality in cardiac translation cases. Indeed, the major cause of late graft failure is graft atherosclerosis.^57^ In this case, a novel, less toxic, and more potent immunosuppressive agent than traditionally used cyclosporine A is developed for survival of the transplanted organ. Fingolimod decreases recirculation of lymphocytes from lymph nodes to inflammatory lesions and graft sites.^55^

Fingolimod exert beneficial effects in transplant recipients beyond immune suppression. Prophylactic administration of FTY720, prolonged the survival of heart allograft transplantation if treatment begin immediately post-transplant.^31^ This graft survival is distinctly dose-dependent from no effect at the lowest dose of 0.3 mg/kg/d to remarkable protection at the highest dose. Indeed, administration of oral capsule of FTY720, prolonged survival; of murine cardiac allograft, as a rescue therapy for acute rejection.^55^ For acute rejection, highest dose of FTY720 is needed. Both early and late graft coronary artery diseases are attenuated using FTY720 treatment. Thus, higher long-term graft acceptance seems achievable using FTY720. Since about of patients who survived 5 years after cardiac transplantation demonstrate significant atherosclerosis on routine coronary angiography, coronary artery disease is considered as a major problem in cardiac transplantation. Graft atherosclerotic lesions are mainly due to immune-mediated damage due to previous acute rejection or persisted immune-mediated reactions in chronic post-transplant phase. Perivascular cuffing and intraluminal accumulation of mononuclear leukocytes, markers of coronary vasculitis, are attenuated using FTY720.^57^ This protection is achieved through dramatic reduction in PBL.^58^

Another mechanism for allograft atherosclerosis is hyperproliferation of smooth muscle cells, but at clinically relevant doses of FTY720, SMC apoptosis has not been observed.^59^ Fingolimod has synergistic effects with calcineurin inhibitors as cyclosporine-A (CsA) and or mammalian target of rapamycin inhibitors as sirolimus.^60^ Of course, the mechanism of action of FTY720 is different from other above mentioned agents. Only mild suppression of interferon-gamma and interleukin-2 production and interference in their function is seen using FTY720 treatment.^57^ Continuous simultaneous application of FTY720 and cyclosporine-A (CsA) abrogate graft atherosclerosis and chronic graft rejection.^2^^,^^57^ For combination therapy, low dose of FTY720 is also effective.^54^ Combined FTY720 plus CsA has been demonstrated to be well-tolerated and suppressed completely development of graft vessel disease.^61^ It allows reduction in CsA dosage in patients with impaired renal/hepatic side-effects.^62^ The efficacy of FTY720 is similar with mycophenolate mofetil (MMF) for prevention of graft atherosclerosis, but less bradycardia seen with FTY compared with MMF.^42^^,^^57^^,^Maintenance mono-therapy with FTY720 modestly ameliorated chronic rejection, but was not enough for treatment of chronic rejection.^63^ Less lymphocytic infiltration has been showed in cardiac allografts by administration of FTY720 for rescuing acute rejection.^57^ Since it does not impair T-cell activation, proliferation and memory response to systemic viral infection it protects grafts from failure without induction of generalized immune suppression and consequent predisposition to infections.^31^ FTY720 treatment was not associated by increased change in rate, nature and severity of infection. Indeed, in the case of severe decrease in leukocyte count, recovery of leukocyte count occurs within only 14 days cessation of FTY720.^64^ The efficacy of FTY720 in human cardiac transplantation should be investigated. As a strong immunosuppressant agent, FTY is shown to be beneficial in the treatment of acute experimental myocarditis without induction of excessive viral replication.^59^

***Vascular Complications of FTY720***

Regarding vascular disorders related to fingolimod, retinal arterial vasospasm and retinal vein occlusion has also been reported.^38^^,^^39^ During platelet activation, S1P is released by platelets.^32^ S1P exerts vasoconstrictive effects on basal arterial tone in isolated arteries through S1P2 and S1P3 receptors. These vasoconstrictive events have been attributed to increased intracellular calcium release and its consequent contractile effects on smooth muscle cells. This induces modest hypertensive effects in long-term. Cerebrovascular constriction is also seen using FTY720 treatment. Interestingly, intramedullary infusion of FTY720 has been shown to prevent hypertensive nephropathy through enhancing sodium excretion as a diuretic agent. It also decrease proteinuria and exert anti-fibrotic effects in renal tissues.^65^

***Effects of FTY720 on Endocrine Functions Related to its Cardiovascular Effects***

FTY720 is suggested to exert potent influence on vascular homeostasis comparable to VEGF, due to the activation of a family protein kinase C (PKC). Since deregulation of S1P1/3 is responsible for cardiac microvascular complications in diabetes, cardiac functions could be improved using FTY720 in this setting via over-expression of S1P1 and enhanced translocation of S1P3.^2^ FTY720 decrease the expression of PKCBII, which is involved in the pathogenesis of diabetic microangiopathy.^30^^,^^31^ This effect is attenuated by expression of PKCBII on endothelial cells. FTY720 might be a beneficial agent for treatment of cardiac microvascular diseases in diabetic patients.^30^ Endothelial cell dysfunction and enhanced endothelial cell permeability seen in the setting of diabetic angiopathy has been improved by application of fingolimod.^60^ Indeed, reduced blood glucose level in diabetic mice has been demonstrated using FTY720 administration. FTY720 showed promising benefits in prevention of T1D related to retention of lymphocytes in the lymph nodes. However, this effect is continued prior to the occurrence of overt hyperglycemia. After disease onset, FTY720 slowed progression of disease.^60^ Sustained endothelial barrier stabilization under hyperglycemic condition is seen by FTY720 application.^53^ It is unknown whether FTY720 is useful in the treatment of metabolic syndrome and its consequent complications. It has not been associated with increased risk of nephrotoxicity, hepatotoxicity, pancreatic toxicity, diabetes, and myelosuppression.^59^ Hyperlipidemia has not yet been reported using FTY720 treatment.

**Figure 1:**
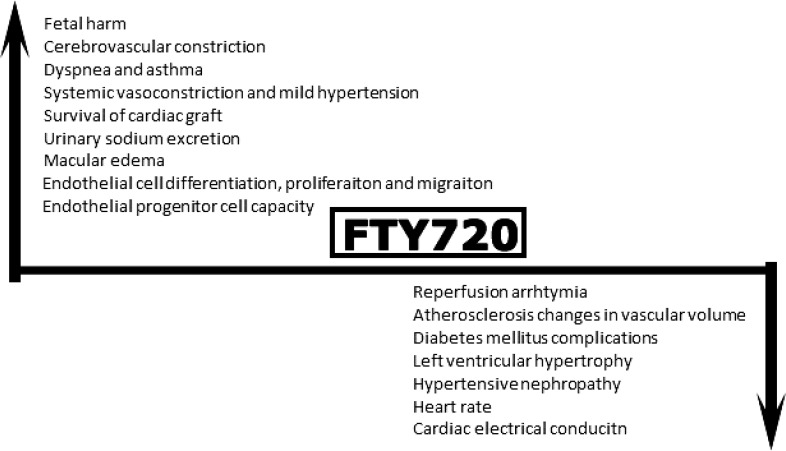
Depicts broad spectrum of cardiovascular manifestations of fingolimod

Figure 1 depicts cardiovascular manifestations of FTY720 briefly. Most surveys on cardiovascular manifestations of fingolimod are performed on volunteers with healthy cardiovascular system, and there is a little guidance on probable side-effects of fingolimod in cases with pre-existing cardiovascular diseases. For cardiac risk stratification, cardiovascular monitoring include one electrocardiogram before the first dose and 6 h post-outpatient hospital cardiology visit at the first day of drug initiation (strict follow-up of blood pressure and heart rate every hour, especially at the first 6 h post-dose).^57^ In patients with cardiovascular risk factors, treatment should be initiated only if benefits outweigh potential. For these cases, adequate patient monitoring is recommended after the first dose.^65^ In cases with compromised cardiac functions, FTY720 is contraindicated.^57^

## Conclusion

Beneficial effects of fingolimod could be higher than its cardiovascular complications through administration of this agent under close observation regarding its side-effects on cardiovascular system. Indeed, we advise clinicians to report all of the cardiovascular manifestations they encounter using FTY.

## Notes

**How to cite this article: **Behjati M, Etemadifar M, Abdar Esfahani M. Cardiovascular effects of fingolimod: A review article. Iran J Neurol 2014; 13(3): 119-26.
